# MDCT of blunt renal trauma: imaging findings and therapeutic implications

**DOI:** 10.1007/s13244-015-0385-1

**Published:** 2015-02-14

**Authors:** M. Bonatti, F. Lombardo, N. Vezzali, G. Zamboni, F. Ferro, P. Pernter, A. Pycha, G. Bonatti

**Affiliations:** 1 Department of Radiology, Bolzano Central Hospital, 5 Boehler St., 39100 Bolzano, Italy; 2 Department of Radiology, University of Verona, Piazzale L.A. Scuro 10, 37134 Verona, Italy; 3 Department of Urology, Bolzano Central Hospital, 5 Boehler St., 39100 Bolzano, Italy

**Keywords:** Acute renal injury, Blunt injury, Computed tomography, Kidney, Trauma

## Abstract

**Objectives:**

To show the wide spectrum of computed tomography (CT) findings in blunt renal trauma and to correlate them with consequent therapeutic implications.

**Methods:**

This article is the result of a literature review and our personal experience in a level II trauma centre. Here we describe, discuss and illustrate the possible CT findings in blunt renal trauma, and we correlate them with the American Association for the Surgery of Trauma (AAST) classification and their therapeutic implications.

**Results:**

CT findings following blunt renal trauma can be grouped into 15 main categories, 12 of them directly correlated with the AAST classification and 3 of them not mentioned in it. Non-operative management, which includes the “watchful waiting” approach, endourological treatments and endovascular treatments, is nowadays widely adopted in blunt renal trauma, and surgery is limited to haemodynamically unstable patients and a minority of haemodynamically stable patients.

**Conclusions:**

The interpretation of CT findings in blunt renal trauma may be improved and made faster by the knowledge of their therapeutic consequences.

***Teaching Points*:**

• *The majority of blunt renal injuries do not require surgical treatment*.

• *CT findings in blunt renal injury must be evaluated considering their therapeutic consequences*.

• *Some CT findings in blunt renal trauma are not included in the AAST classification*.

## Introduction

Renal injuries represent a relatively common event, involving 8–10 % of the patients admitted to an emergency department because of abdominal trauma, and the incidence is increasing parallel to the increase in motor-vehicle accidents [1–4]. In 80–95 % of the cases renal injuries occur in the setting of blunt trauma, as a result of a direct trauma to the back, flank, lower thorax or upper abdomen or of a rapid deceleration, whereas penetrating traumas are responsible for the remaining 5–20 % [3, 5–7]. Renal trauma may occur in isolation or in association with other visceral injuries, with the majority of isolated renal injuries represented by low-grade lesions [8–10].

Although gross haematuria is the typical presenting symptom of renal injury, it may be absent in about 5 % of renal injuries, and its presence is not directly correlated with injury severity: for example, haematuria is typically absent in vascular pedicle injuries and ureteral-pelvic injuries [11, 12].

According to the American Association for the Surgery of Trauma (AAST), on the basis of surgical findings renal injuries can be subdivided into five main categories (Table 1), with a progressively poorer prognosis [13]. The suggested treatment options, according to the AAST injury grade, in haemodynamically stable patients affected by renal injuries are reported in Table 2. In the past decades, the indications for surgical treatment of renal trauma have progressively decreased with a consequent drastic reduction in nephrectomy rates, and, nowadays, non-operative management (NOM), which includes the simple watchful waiting (“wait-and-see”) strategy, endovascular treatments and endourological treatments, can be considered the most appropriate first line approach for about 90–95 % of renal injuries [14–20]. On the other hand, renal exploration remains mandatory in every case of haemodynamic instability following renal trauma and if a pulsatile or expanding retroperitoneal haematoma is identified during exploratory laparotomy performed for other abdominal injuries.

**Table 1:** American Association for the Surgery of Trauma (AAST) classification of renal trauma

Grade	Type	Description
I	Parenchyma Haematoma	Microscopic or gross haematuria with normal urologic studies (contusion) Non-expanding subcapsular haematoma
II	Parenchyma Haematoma	Laceration <1 cm in depth, without collecting system rupture Non-expanding perirenal haematoma confined to retroperitoneum
III	Parenchyma	Laceration >1 cm in depth without collecting system rupture
IV	Parenchyma Vascular	Laceration with collecting system rupture Main renal artery/vein injury with contained haemorrhage
V	Parenchyma Vascular	Shattered kidney Avulsion of renal hilum that devascularises kidney

**Table 2:** Treatment options in haemodynamically stable patients affected by renal injury (according to the AAST classification)

Grade	Type	Treatment
I-III	Every	NOM wait and see
IV	Parenchyma Vascular	NOM endourological Debated, surgical vs. NOM interventional radiological
V	Parenchyma Vascular	Debated, NOM is increasingly adopted Surgical. NOM endourological in case of partial pielo-uretheral lacerations
???	Vascular	NOM interventional radiological

*NOM* non-operative management, ??? = lesions not included in the AAST classification

## Indications for renal imaging and imaging modalities

In case of blunt thoraco-abdominal trauma, a clear distinction must be made between haemodynamically unstable and haemodynamically stable or stabilised patients; indeed, the former must immediately undergo surgical exploration in order to try to improve their outcome, whereas the latter should undergo imaging studies in order to better assess the presence of visceral injuries and guide treatment. Although some controversies still exist, in haemodynamically stable (or stabilised) patients who have suffered blunt thoracic and/or abdominal trauma, absolute indications for imaging in the suspicion of renal injuries are gross haematuria, microscopic haematuria associated with hypotension, suspicion of major injuries to other viscera and trauma dynamics strongly associated with renal injury (i.e., rapid deceleration, fall from great height and direct contusion to the flank) [3, 5].

Several different imaging techniques have been considered as the initial imaging tool in the suspicion of renal injury in haemodynamically stable patients; nowadays, however, thanks to its wide availability, reproducibility, scan speed and high spatial resolution, contrast-enhanced multidetector computed tomography (MDCT) is considered the gold standard imaging modality in case of suspicion of renal injury and has completely replaced intravenous urography for this purpose [3, 5, 21–24]. The great advantage of MDCT is its ability to assess, in an extremely rapid scanning time, the presence of lesions of the parenchyma, vessels and collecting system and of injuries to other abdominal organs.

## MDCT of renal trauma: technique and protocols

Multidetector CT systems are nowadays ubiquitously available in trauma centres and represent the minimum requirement for emergency renal imaging. The availability of modern scanners with a high number of detector rows (16 or more) enables shortening acquisition times, therefore reducing the possibility of motion artefacts and increasing image quality.

A correct positioning of the patient on the CT table represents a fundamental prerequisite for performing high-quality MDCT studies. Whenever possible, the patient should lay supine on the table, with the arms bent over the head in order to prevent beam-hardening artefacts; if that position cannot be obtained, the patient’s arms should be placed flexed on a large pillow ventrally to the patient’s chest [25]. Moreover, all the medical devices that are not strictly necessary should be removed from the scan area.

The MDCT scanning protocol in the setting of suspected renal trauma should always be always tailored according to the clinical conditions of the patient. Most authors sustain that a non-enhanced acquisition should always be performed in order to detect intraparenchymal, subcapsular or perinephric haematomas, which will appear as slightly hyperattenuating areas or collections during this phase, whereas they might be barely recognisable on post-contrast images [26–28]. According to our experience, however, the performance of native scans is not necessary in the setting of renal trauma for two reasons: first, the large majority of the above-mentioned lesions are clearly recognisable also on post-contrast images; second, those lesions that might eventually be missed (i.e., small intraparenchymal or subcapsular haematomas) do not have any therapeutic consequences. In any case, when acquiring post-contrast images using a dual-energy technique, virtual non-enhanced images may be retrospectively generated from post-contrast ones by means of commercially available software and show similar accuracy to true non-contrast ones for the detection of haematomas [29].

On the other hand, post-contrast acquisitions must always be performed in case of suspicion of renal injuries. About 1.7–1.5 ml/kg of 350–370 mgI/ml iodinated contrast material must be administered using a flow rate of at least 3.5 ml/s, followed by a 50-ml saline flush. Contrast-enhanced scans should be acquired cranio-caudally, with thin collimation (0.625 mm). Multi-planar reformats (MPR), maximum intensity projection (MIP) and volume-rendering (VR) reconstructions represent extremely useful tools for imaging interpretation. According to the wide variability of circulatory conditions in trauma patients, post-contrast scans should be always performed using a bolus-tracking technique. A late arterial/corticomedullary phase acquisition should be acquired with a 15-s delay from a 100-HU peak aortic enhancement when using a bolus-tracking technique or with a 30-s fixed delay after injection if bolus-tracking is not available; this phase is mandatory for recognising arterial vascular lesions (i.e., renal artery avulsion or thrombosis, pseudoaneurysms and artero-venous fistulae) and for detecting active arterial contrast material extravasation; moreover, it enables to accurately depict the arterial vascular anatomy and eventual variants. The portal-venous or nephrographic phase, acquired 70-80 s after the start of contrast material injection, represents the best phase for detecting parenchymal lesions (i.e., lacerations and segmental infarctions), venous vascular lesions (i.e., avulsion or thrombosis of the renal vein) and haematomas and should be always performed in case of suspicion of renal injury; moreover, it represents a useful aid for assessing the relevance of an active arterial contrast material extravasation and enables accurately ruling out injuries to other parenchymatous abdominal organs. The decision to acquire a delayed excretory scan is strictly dependent on the patient’s clinical conditions and the findings in the previous phases. The timing of the delayed acquisition depends on renal function and may vary from 5 to 15 or more minutes; 8 min may represent a good compromise in the majority of the cases. Delayed acquisitions are fundamental for the detection of collecting system injuries, by showing hyperdense urine extravasation, and may also help in the differential diagnosis between renal contusion and infarction [3].

## MDCT findings and therapeutic consequences

MDCT can generate a wide spectrum of findings in renal trauma and the majority of these can be easily categorised into the above-mentioned five AAST grades; moreover, MDCT may also show some findings that are not mentioned in the AAST classification, the latter being based on the kidney appearance at surgery. The AAST classification must be kept in mind when reporting renal injuries, particularly because of its prognostic implications; in any case, subtle differences in AAST grade do often not have practical consequences for patient management. In the following paragraphs we will describe the possible MDCT findings in renal trauma grouped by their therapeutical management and not simply by the AAST grading scale.

### Non-operative management: “wait-and-see”

**Fig. 1:**
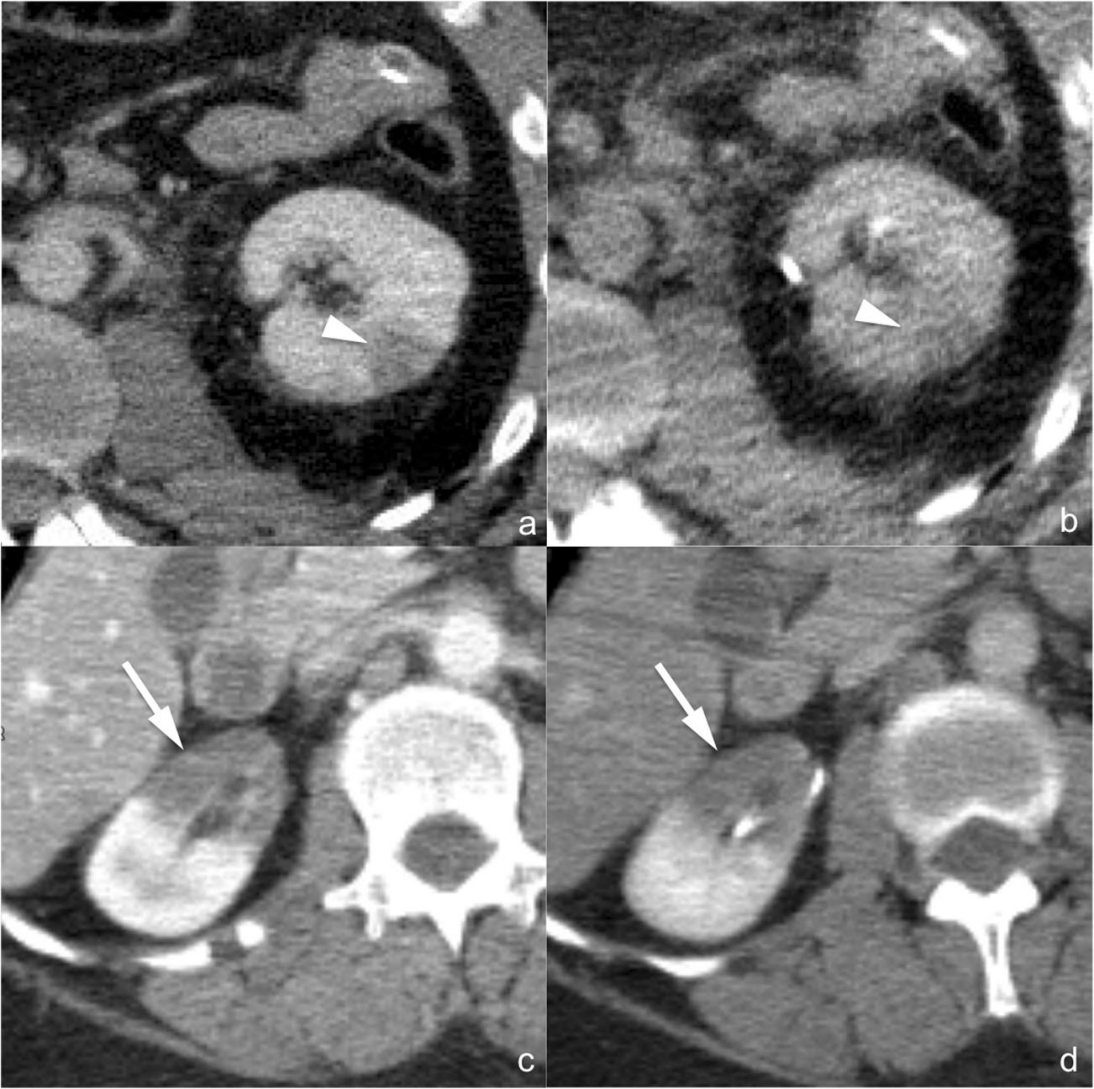
**a**–**d** Renal contusion (AAST grade I) (**a** and **b**) and renal infarction (AAST grade IV) (**c** and **d**). Axial multiplanar reconstruction (*MPR*) images (1.5 mm thick **a** and **b**; 3 mm thick **c** and **d**) acquired during the nephrographic phase (**a** and **c**) and the 8-min delayed phase (**b** and **d**) of the study in two different patients. Renal contusion appears as an ill-defined, irregularly shaped area of reduced contrast enhancement during the nephrographic phase (**a**, *arrowhead*) and tends to isodensity in the delayed phase (**b**, *arrowhead*), whereas segmental infarction appears as a wedge-shaped, sharply defined area of reduced/absent contrast enhancement during the nephrographic phase (**c**, *arrow*) and shows marked hypodensity also in the delayed phase (**d**, *arrow*)
- *Subcapsular haematoma* (*AAST grade I*). Subcapsular haematoma (Fig. 2a–b) appears as a well-delimited, non-enhancing fluid collection located between the renal parenchyma and the renal capsule. Depending on its size, the collection may show a crescent or a biconvex shape; in the latter case, a mass effect is usually exerted on the adjacent parenchyma, which may become flattened or indented. The attenuation of the collection varies according to its age, and hyperdensity may be seen if blood clots are present. To be classified as a grade I lesion, a subcapsular haematoma must not be associated with parenchymal lacerations and no active contrast material extravasation should be observed; indeed, subcapsular haematomas are often associated with higher grade parenchymal lesions. Moreover, if a capsular lesion coexists, the haematoma often extends to the perirenal space (Fig. 2b). A simple “wait-and-see” management is indicated in case of subcapsular haematoma, and no follow-up imaging studies are required if the clinical and laboratory data remain stable [30–32].

- *Parenchymal contusion* (*AAST grade I*). Parenchymal contusions (Fig. 1a–b) appear as ill-defined, irregularly shaped areas of decreased and delayed contrast enhancement within the renal parenchyma. On non-enhanced images their density varies according to the time elapsed from the traumatic event, the patient’s haematocrit and the coagulation status: typically they appear isoattenuating to the adjacent cortex, but hyperattenuation may also be observed if blood clots are present. The corticomedullary phase is not useful for identifying renal contusions because of the physiological heterogeneity of parenchymal enhancement. On the other hand, in the majority of the cases renal contusions are clearly recognisable during the nephrographic phase in which they usually appear slightly hypodense in comparison to the adjacent parenchyma; however, a substantial isodensity might be found if blood clots are present. On delayed acquisitions, contusions show persistent contrast retention, and this characteristic may be helpful for differentiating them from renal infarctions (Fig. 1c–d) that remain hypodense. A simple “wait-and-see” management is indicated in case of renal contusions, and no follow-up imaging studies are required if the clinical and laboratory data remain stable [30–32].

**Fig. 2:**
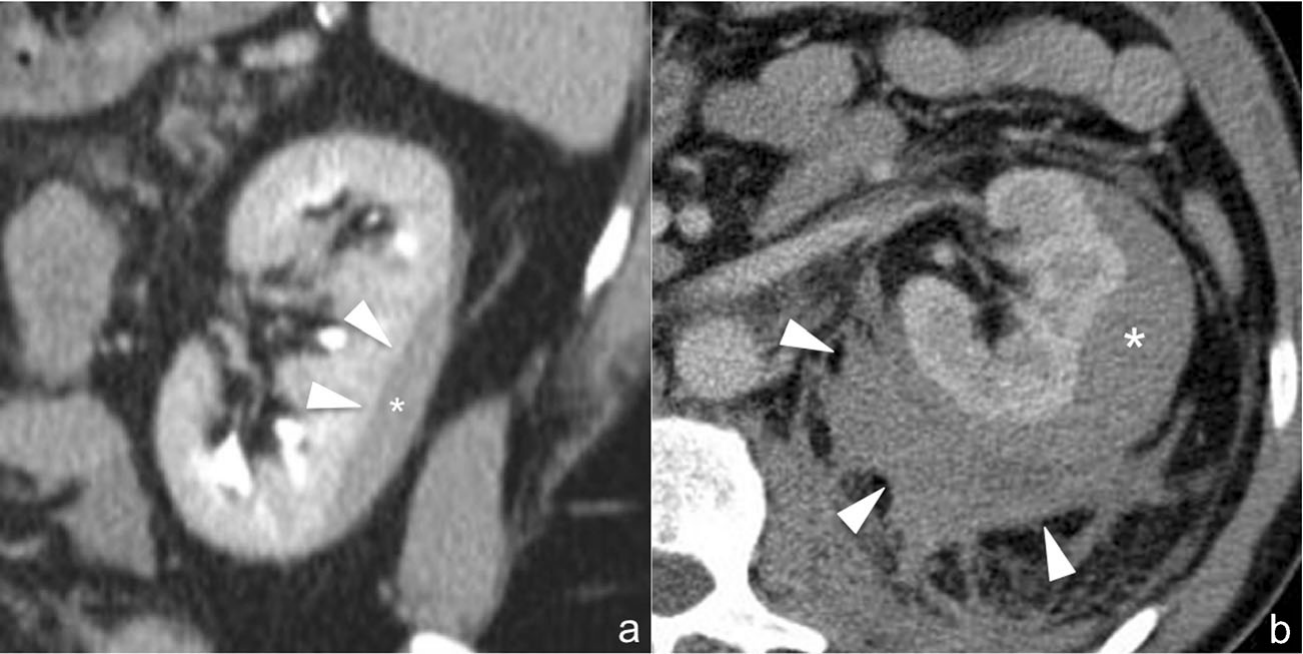
**a**–**b** Subcapsular haematoma (AAST grade I) (**a**) and perirenal haematoma (AAST grade II) (**b**). The 3-mm-thick multiplanar reconstruction (*MPR*) coronal image (**a**) acquired during the delayed phase of the study shows a well-delimited hypodense biconvex-shaped collection (*star*), located between the renal parenchyma and renal capsule, which determines the mild mass effect on the adjacent parenchyma (*arrowheads*), representing subcapsular haematoma. A similar finding may also be observed in the 3-mm-thick multiplanar reconstruction (*MPR*) axial image (**b**) acquired during the nephrographic phase in another patient in which, however, an irregularly delimited inhomogenously hypodense collection, representing perirenal haematoma (*arrowheads*), is appreciable between the renal parenchyma and the Gerota fascia. In both cases neither parenchymal lacerations nor active contrast material extravasations can be observed
- *Perirenal haematoma* (*AAST grade II*). Perirenal haematoma (Fig. 2b) appears as a poorly delimited, non-enhancing fluid collection located in the fatty area between the renal capsule and Gerota fascia. Its attenuation varies according to the age, appearing inhomogenously hyperattenuating in the acute/subacute stage and becoming progressively iso- or hypoattenuating in the chronic stage. Perirenal haematoma usually does not cause a mass effect on the adjacent renal parenchyma, but large haematomas may displace the kidney and nearby bowel and may also cross the midline, ventrally to the aorta and inferior vena cava, invading the contralateral perirenal space. An extension to the pelvis may also be observed in large perirenal haematomas. Although perirenal haematomas may represent isolated lesions, in most cases they are associated with parenchymal lacerations and the presence of opacified urine extravasation must be accurately ruled out by means of delayed scans. Moreover, particular attention must be paid to collections located medially to the renal hilum: in these cases, the presence of vascular pedicle and uretero-pelvic junction lesions must be ruled out accurately. The tiny perinephric strands that may be observed in patients with impaired renal function should not be misdiagnosed as perirenal haematomas: indeed, these strands are homogeneously hypodense, bilateral and surround the entire kidneys. In case of isolated perirenal haematomas, a simple “wait-and-see” management is indicated and no follow-up imaging studies are required if the clinical and laboratory data remain stable [30–32].
- *Parenchymal laceration not involving the collecting system* (*AAST grade II-III*): Parenchymal lacerations (Fig. 3a–b) appear as irregular or linear clefts of absent contrast enhancement, starting from the renal surface and extending deep into the renal parenchyma, and they are best depicted during the nephrographic phase. Their attenuation varies according to their age and the presence of blood clots, and they are usually associated with perirenal haematomas. If a parenchymal laceration is present, delayed acquisitions should be performed in order to rule out urine extravasation (Fig. 3b). Moreover, it is extremely important to exclude the presence of active contrast material extravasation. The AAST subdivides parenchymal lacerations into grade II if they extend less than 1 cm in depth and grade III if they extend more than 1 cm; in any case, there is no difference in the treatment of grade II and III lesions, which must follow the simple “wait-and-see” management. No follow-up imaging studies are required if the clinical and laboratory data remain stable [30–32].

**Fig. 3:**
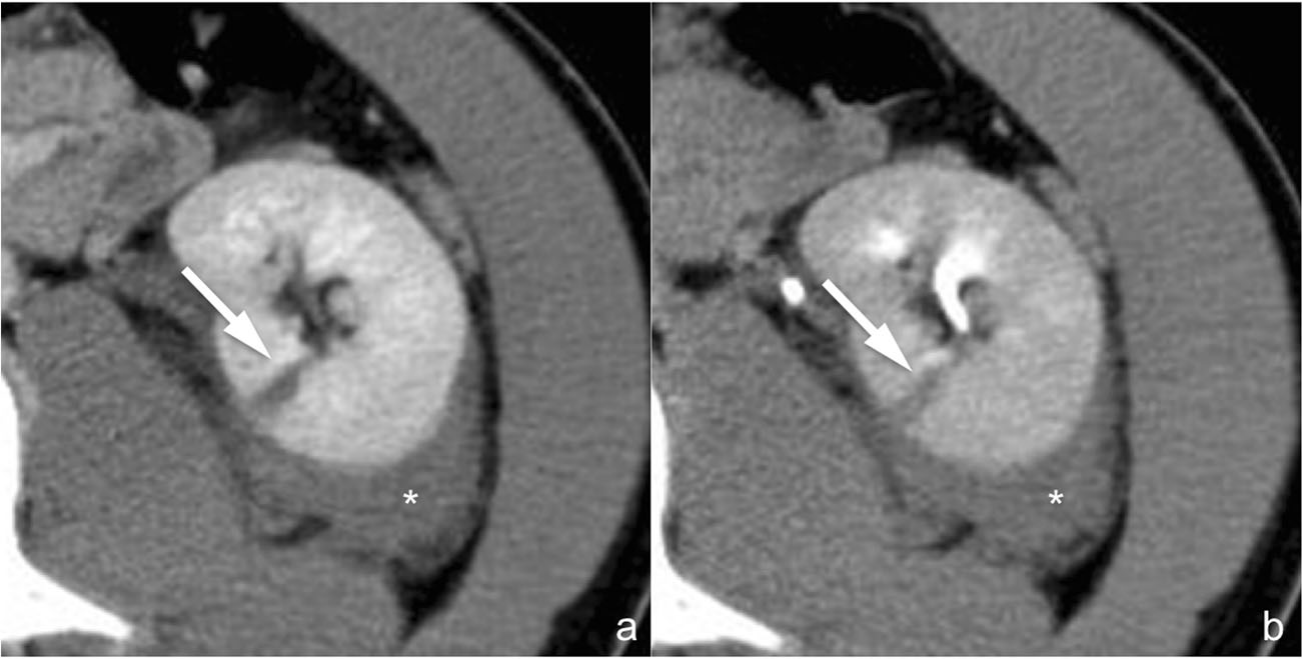
**a**–**b** Parenchymal laceration not involving the collecting system (AAST grade II–III). On these 3-mm-thick multiplanar reconstruction (*MPR*) axial images acquired during the nephrographic (**a**) and 8-min delayed phase (**b**) of the study in the same patient, a linear cleft of absent contrast enhancement (*arrow*) starting from the renal surface and extending deep into the renal parenchyma is clearly appreciable, representing parenchymal laceration. On delayed image (**b**), no lack of hyperdense urine can be seen, excluding collecting system rupture. A perirenal haematoma (*star*) coexists posteriorly to the kidney
- *Segmental infarction* (*AAST grade IV*). Segmental infarctions (Fig. 1c–d) appear as sharply demarcated, wedge-shaped areas of absent contrast enhancement, showing a subcapsular basis and apex directed to the hilum, and are the consequence of dissection and/or thrombosis of segmental vessels. Sharp margins and lack of enhancement during the delayed phase are the characteristics that must be identified in order to perform a differential diagnosis with renal contusions (Fig. 1a–b). The coexistence of multiple infarctions is quite frequent. A simple “wait-and-see” management is indicated in the majority of cases, whereas surgical debridement is indicated in case of infarctions involving more than 50 % of the renal parenchyma. No follow-up imaging studies are required if the clinical and laboratory data remain stable [30–32], whereas a CT scan is mandatory in case of pain development in order to exclude colliquation or abscess formation, findings that imply surgical exploration.
- “*Shattered kidney*” (*AAST grade V*). The term “shattered kidney” is used to describe a kidney ruptured in multiple fragments by severe and deep lacerations (Fig. 4a–b). Renal fragments are usually surrounded and subdivided by a fluid collection that occupies the entire renal loggia; some parenchymal fragments may appear devitalised or non-enhancing because of traumatic occlusion of their arterial supply. Particular attention must be paid to excluding the presence of active contrast material extravasation and urine leakage: indeed, if these are ruled out, an increasing number of reports suggest that haemodynamically stable patients can be safely treated conservatively by means of a simple “wait-and-see” treatment, whereas surgical exploration should be reserved for haemodynamically unstable patients and endovascular and/or endourological treatments for patients showing blood and/or urine extravasation [16, 17, 20, 33–38]. Due to the severity of the injury, follow-up CT studies might represent a reasonable option in these patients, also in case of clinical and laboratory stability, in particular in order to detect vascular complications early, such as pseudoaneurysms and artero-venous fistulae, which might have life-threatening effects in an acute setting.

**Fig. 4:**
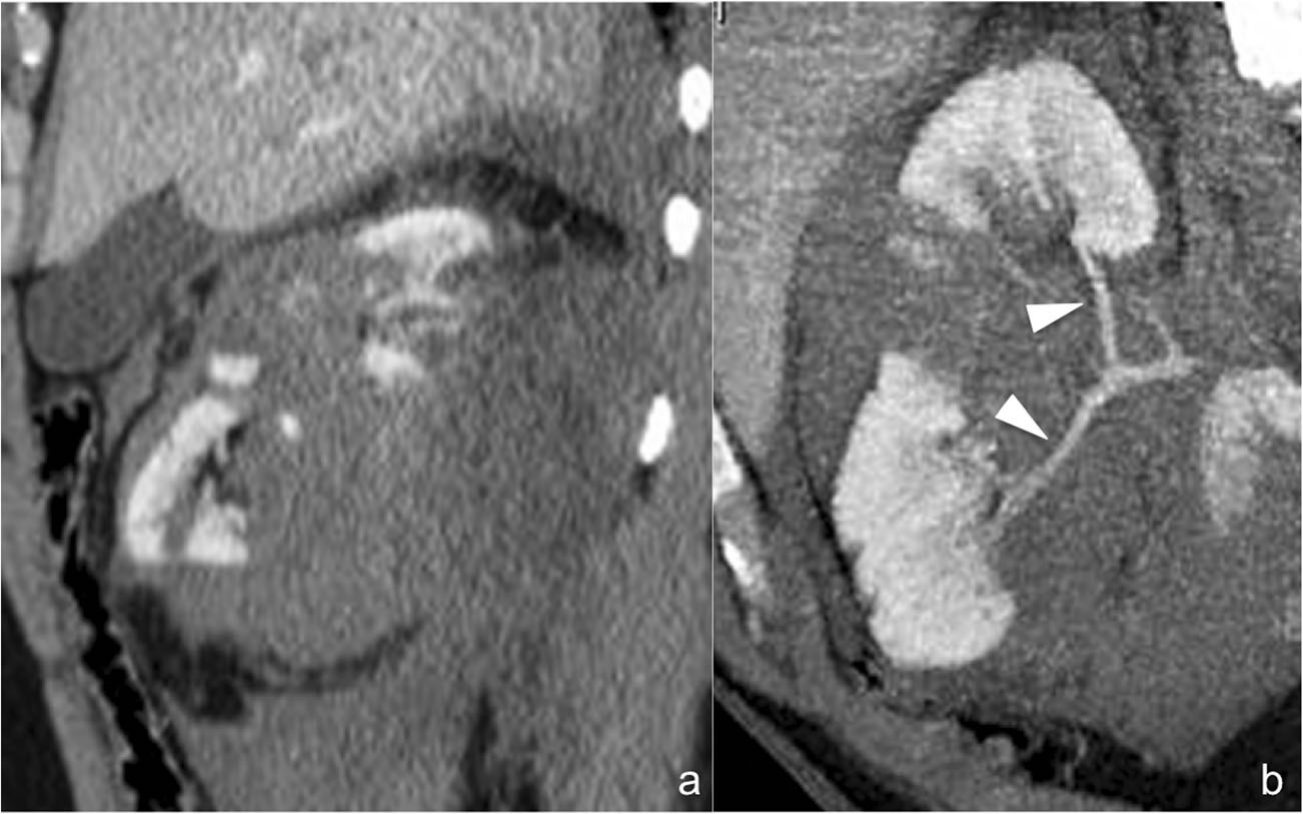
**a**–**b** Shattered kidney (AAST grade V). The 3-mm-thick multiplanar reconstruction (*MPR*) coronal image (**a**) acquired during the nephrographic phase of the study shows a large inhomogenously hypodense collection occupying the renal and perirenal spaces with multiple vascularised kidney fragments within, representing a so-called shattered kidney. On the 10-mm-thick para-coronal maximum intensity projection (*MIP*) of the same series (**b**), the main branches of the *right* renal artery (*arrowheads*) can be accurately delimited and no active contrast material extravasation can be observed

### Non-operative management: endourological treatment

**Fig. 5:**
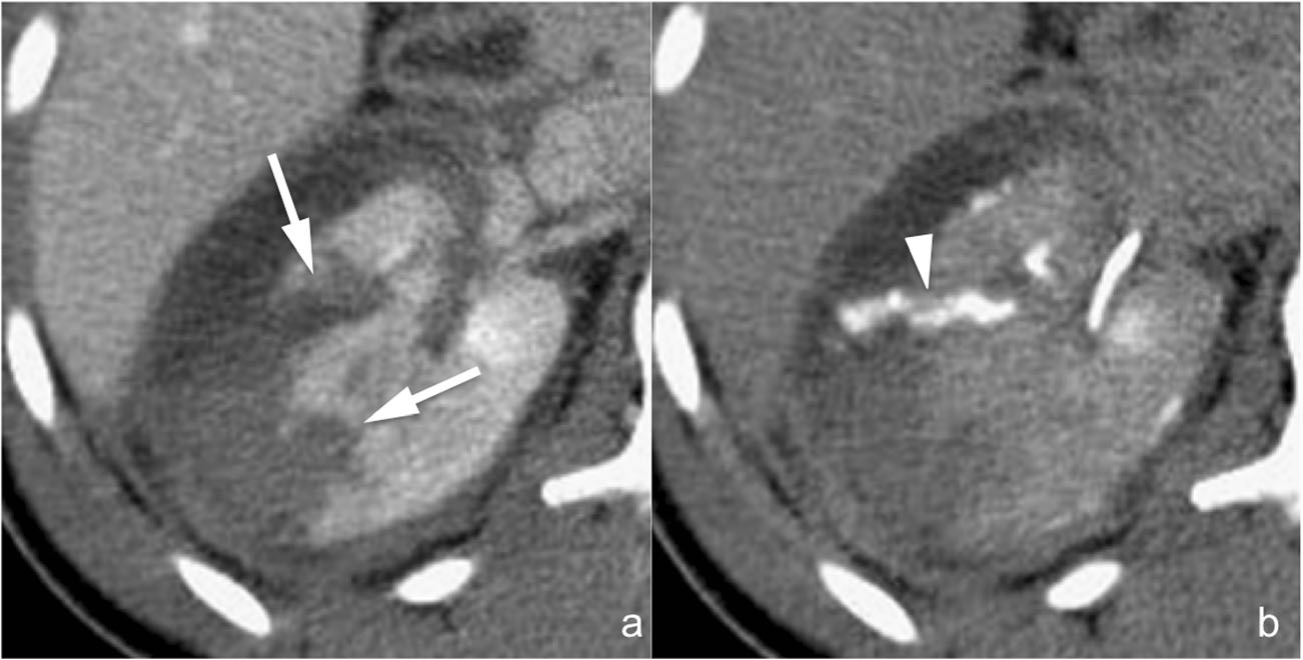
**a**–**b** Parenchymal laceration involving the collecting system (AAST grade IV). On these 3-mm-thick multiplanar reconstruction (*MPR*) axial images acquired during the nephrographic (**a**) and 8-min delayed phase (**b**) of the study in the same patient, multiple irregular clefts of absent contrast enhancement (*arrows*), starting from the renal surface and extending deep into the renal parenchyma, are clearly appreciable, representing parenchymal lacerations. On delayed image (**b**), hyperdense urine leakage (*arrowhead*) is recognisable as a sign of collecting system rupture
- *Ureteropelvic junction laceration* (*AAST grade V*). Ureteropelvic junction laceration (Fig. 6a–b) is usually a consequence of fast deceleration traumas and is associated with a large perirenal fluid collection, mostly located medially to the renal hilum and often extending caudally over the psoas. Renal contrast material excretion is normal and the calyceal system is usually intact, while hyperdense urine extravasation can be highlighted in the delayed phase at the renal hilum. Ureteropelvic junction avulsion, which otherwise shows the same CT findings (Fig. 6c–d), can be excluded if hyperdense urine is seen in the downstream ureter (Fig. 6b). Ureteral stenting is the treatment of choice for ureteropelvic junction laceration, and at least one follow-up CT examination is indicated in order to assess urinoma evolution and detect any complications early; attention must be paid to the proximal extremity of the JJ catheter, which should be located within the upper calyceal group.

- *Parenchymal laceration involving the collecting system* (*AAST grade IV*). Parenchymal lacerations involving the collecting system (Fig. 5a–b) extend deep into the medulla and are univocally associated with perirenal fluid collections secondary to blood and urine accumulation. Urine extravasation can be highlighted during the delayed phase of the study as markedly hyperdense material within the perirenal haematoma (Fig. 5b). Ureteral stenting is the treatment of choice in collecting system injuries and at least one follow-up CT examination is indicated in order to assess the evolution of the urinoma and to detect eventual complications early, such as superinfection; attention must be paid to the proximal extremity of the JJ catheter, which should be located within the upper calyceal group in order to maximise its effectiveness in urine drainage.

**Fig. 6:**
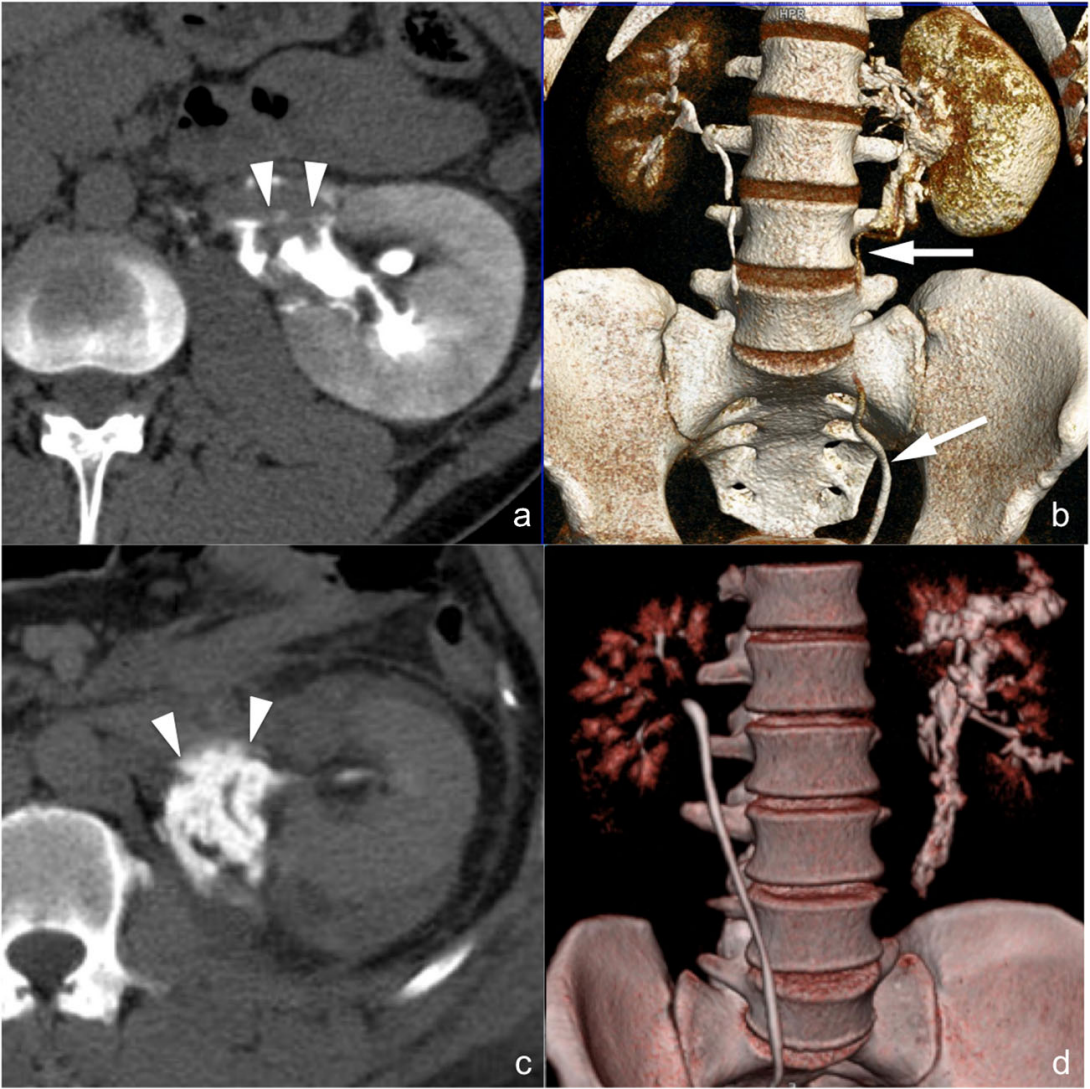
**a**–**d** Laceration of the ureteropelvic junction (**a** and **b**) and avulsion of the ureteropelvic junction (**c** and **d**) (AAST grade V both). On the 3-mm-thick multiplanar reconstruction (*MPR*) axial images acquired during the delayed phase of the study in two different patients (**a** and **c**) a hyperdense urine leakage can be observed medially to the kidney hilum (*arrowheads*), a finding suspicious for a ureteropelvic junction lesion. The volume-rendering technique (*VRT*) reconstructions of the same series (**b** and **d**) show that, in the first case (**b**), the downstream ureter is opacified by hyperdense urine, therefore excluding a complete ureteropelvic disconnection, whereas in the second case (**d**), no opacified urine can be observed in the downstream ureter, indicating therefore a complete ureteropelvic avulsion

### Non-operative management: interventional radiology

**Fig. 7:**
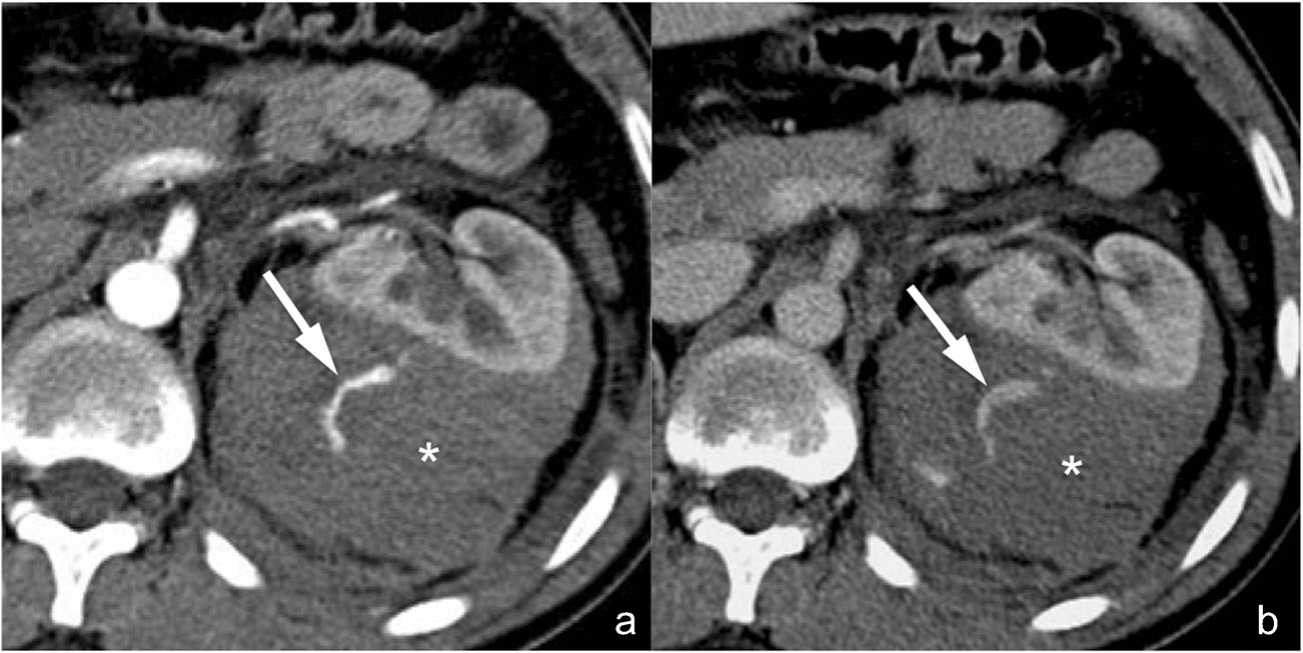
**a**–**b** Active arterial contrast extravasation from a capsular artery (lesion not included in the AAST classification). On these 3-mm-thick multiplanar reconstruction (*MPR*) axial images acquired during the arterial (**a**) and nephrographic (**b**) phases of the study in the same patient, a large perirenal haematoma (*star*) determining a ventral dislocation of the kidney is clearly recognisable. No parenchymal lacerations can be observed, but an active contrast material extravasation (*arrow*) is recognisable within the haematoma during the arterial phase, showing a mild increase in size during the nephrographic phase as a consequence of a capsular artery rupture
- *Arterial pseudoaneurysm* (*not included in the AAST classification*). Arterial pseudoaneurysm (Fig. 8a–b) appears as a well-delimited ovoid lesion located within the renal parenchyma, enhancing as much as the adjacent arteries during the arterial phase and not increasing in size or density during the subsequent phases in which, indeed, it becomes isoattenuating to the blood pool; these characteristics enable a differential diagnosis with active contrast material extravasation. Digital subtraction angiography (Fig. 8c) with superselective arterial embolisation represents the treatment of choice in order to prevent delayed rupture; surgery may be limited to the few cases in which haemostasis and arterial wall defect repair are required [42].

- *Active contrast material extravasation from branching renal arteries* (*not included in the AAST classification*). Active contrast material extravasation from peripheral arterial branches (Fig. 7a–b) is not directly mentioned in the AAST classification of renal trauma. At CT, small arterial bleedings appear as poorly delimited contrast material extravasations, sometimes flame-shaped, usually located in the setting of a perirenal haematoma, that appear hyperdense in the arterial phase and increase in quantity and density in the following phases. Active arterial contrast material extravasation is often associated with high-grade parenchymal lesions, but occasionally it may also occur in low-grade ones. Non-operative management is indicated in haemodynamically stable patients if no other lesions requiring surgery are detected at CT, and digital subtraction angiography with superselective arterial embolisation represents the treatment of choice [39, 40]; angioembolisation might be avoided in patients with small perirenal haematomas and minimal signs of active bleeding [41]. A CT follow-up should be considered after angioembolisation in order to detect eventual complications, such as pseudoaneurysms and large ischaemic events.

**Fig. 8:**
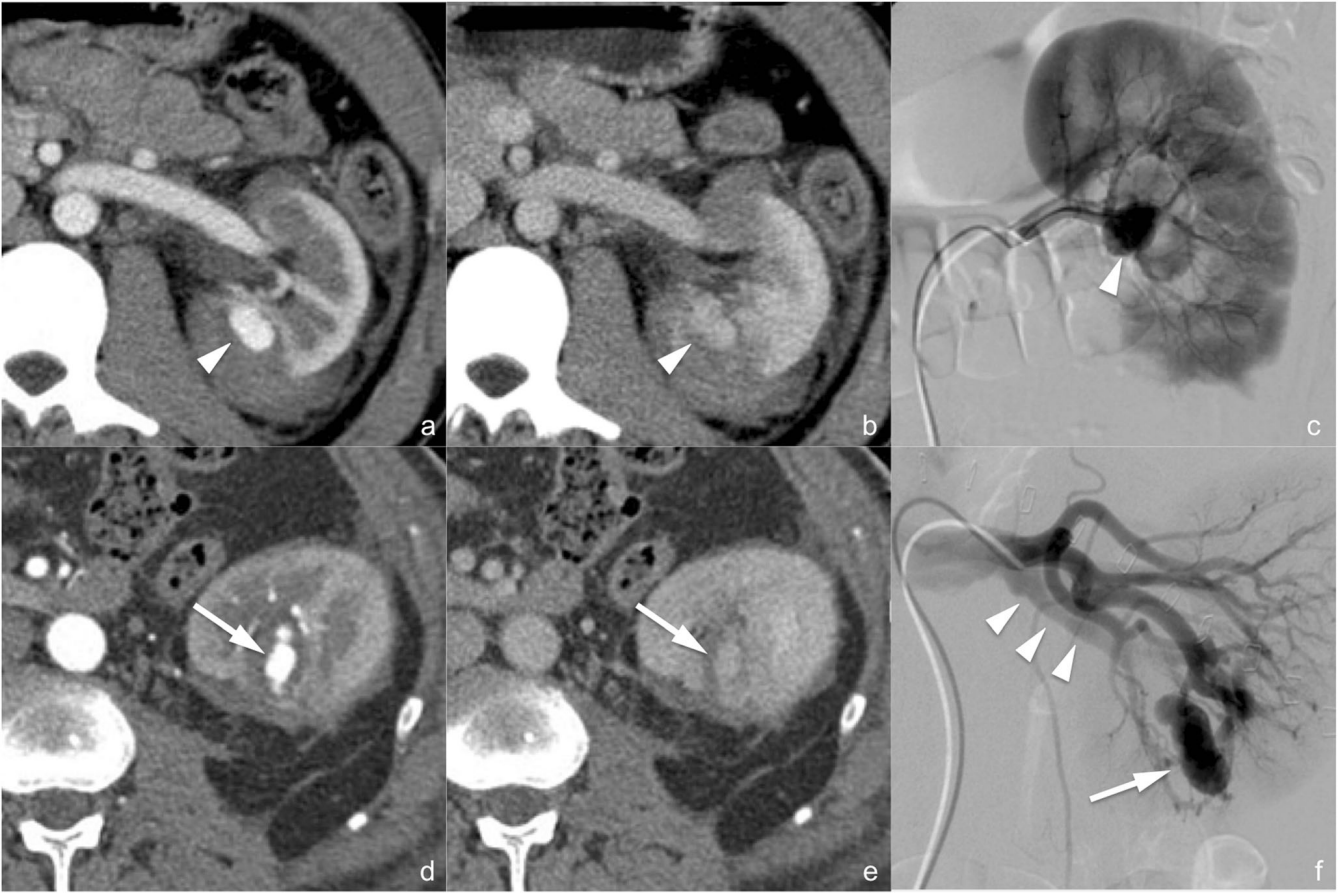
**a**–**f** Arterial pseudoaneurysm and artero-venous fistula (lesions not included in the AAST classification). On the 3-mm-thick multiplanar reconstruction (*MPR*) axial images acquired during the arterial (**a** and **d**) and nephrographic (**b** and **e**) phases of the study in two different patients, a well-delimited oval lesion (*arrowheads* in the first patient and *arrows* in the second one) with a density that varies parallel to the density of the arterial vessels is clearly recognisable. Digital subtraction angiography characterised these lesions as an arterial pseudoaneurysm in the first patient (**c**) and as an artero-venous fistula in the second one (**f**) in which an early venous output (*arrowheads*) was recognisable
- *Artero-venous fistula* (*not included in the AAST classification*). Artero-venous fistulae (Fig. 8d–e) usually have the same CT appearance as arterial pseudoaneurysms. An important finding that enables the differential diagnosis between the two lesions is the identification of a dilated renal vein that enhances early during the arterial phase of the study; anyway, a true arterial phase should be acquired in order to appreciate this finding that might be obscured by the physiological venous return during the corticomedullary phase. Digital subtraction angiography (Fig. 8f) with superselective arterial embolisation represents the treatment of choice in order to prevent delayed rupture and arterial hypertension development.

### Surgical management

**Fig. 9:**
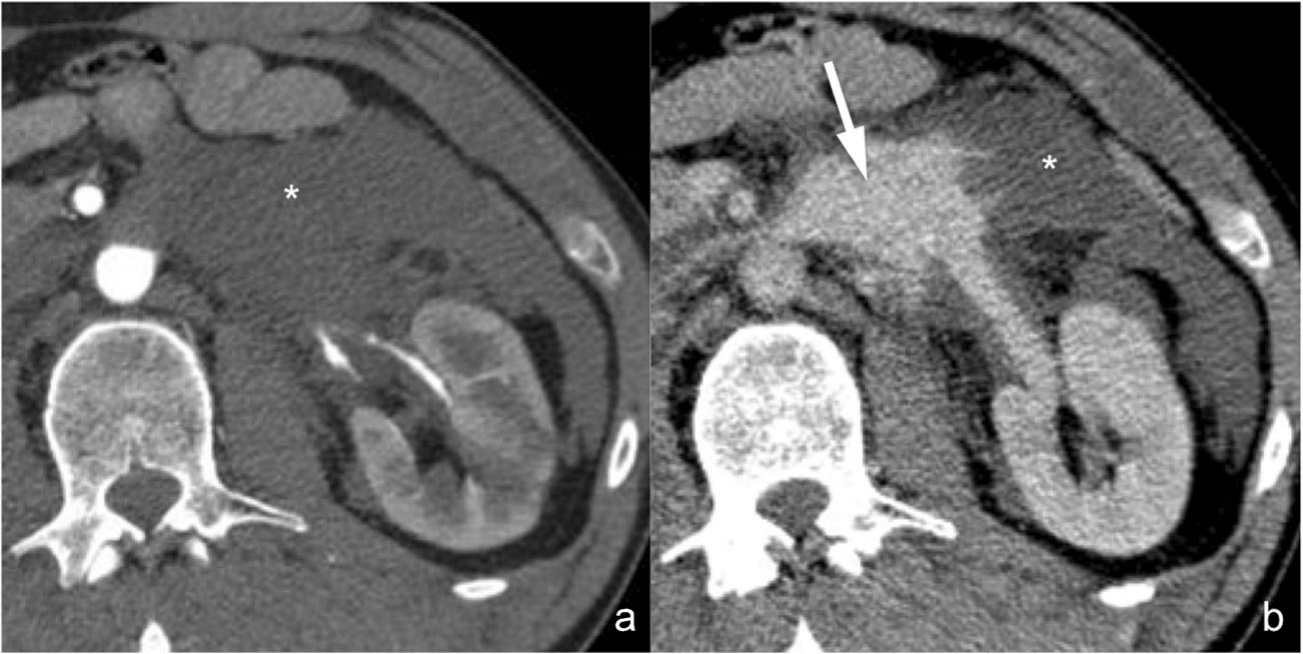
**a**–**b** Main renal vein laceration (AAST grade V). On these 3-mm-thick multiplanar reconstruction (*MPR*) axial images acquired during the arterial (**a**) and nephrographic (**b**) phases of the study in the same patient, a large perirenal-pararenal haematoma (*star*) is recognisable medially-ventrally to the kidney. No active contrast material extravasation can be observed during the arterial phase of the study, whereas a large contrast material extravasation (*arrow*) appears ventrally to the renal vein in the subsequent nephrographic phase, representing renal vein laceration
- *Avulsion of the ureteropelvic junction* (*AAST grade V*). CT findings in case of complete ureteropelvic junction transection (Fig. 6c–d) are the same as in case of ureteropelvic junction laceration, with the only important difference a complete absence of opacified urine in the downstream ureter (Fig. 6d). Differently than in case of ureteropelvic junction laceration, ureteropelvic junction avulsion requires surgical repair because endourological treatments are usually ineffective.

- *Avulsion of the main renal artery or vein* (*AAST grade V*). Avulsions of the main renal artery or vein (Fig. 9a–b) represent extremely dangerous lesions that, in the majority of cases, preclude the patient to undergo CT because of haemodynamic instability. Both lesions are associated with large perirenal haematomas, prevalently located medially to the renal hilum, with abundant contrast material extravasation between the kidney and the aorta in the arterial and/or portal venous phase. Renal artery avulsion in the majority of the cases results in a completely or at least largely devascularised kidney, whereas in case of renal vein avulsion renal enhancement is usually delayed and reduced, but uniformly present. Vascular pedicle avulsion represents a life-threatening emergency: no delayed CT scans must be performed and immediate surgical exploration is mandatory.

### Debated management

**Fig. 10:**
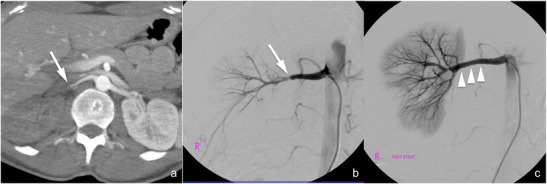
**a**–**c** Main renal artery dissection/thrombosis. The 10-mm-thick maximum intensity projection (*MIP*) axial image acquired during the arterial phase of the study (**a**) shows a hypovascularised right kidney as a consequence of renal artery dissection (*arrow*). The finding was confirmed at subsequent digital subtraction angiography (**b**, *arrow*) in which a residual flow was appreciable downstream from the dissection; a stent was placed (**c**, *arrowheads*) with good flow recovery
- *Injuries to the main renal artery or vein with a contained haematoma or partial vessel laceration* (*AAST grade IV*). This type of injury manifests with the presence of a perirenal haematoma associated with active arterial or venous bleeding from hilar vessels (Fig. 11). The haematoma is usually located medially to the renal hilum and may extend caudally along the psoas; the kidney is often displaced laterally. Both surgical and endovascular treatments may be considered, also according to the centre’s experience, with the latter associated with lower nephrectomy rates [16].

- *Thrombosis of the main renal artery or vein* (*AAST grade IV*). Arterial thrombosis is usually secondary to traumatic dissection of the main renal artery (Fig. 10a–c), and the resulting occlusion may be complete or partial; consequently, the kidney may appear completely devascularised or hypovascularised. The thrombus, when detectable, appears as an endovascular filling defect, hyperdense on non-enhanced images and hypodense in post-contrast scans. Main renal vein thrombosis is rare and causes delayed enhancement of the kidney in comparison with contralateral renal enlargement and parenchymal oedema. Management of these injuries is extremely controversial: in particular, in case of renal artery dissection both surgical and endovascular revascularisations (Fig. 10b–c) have shown good results [43, 44] and the treatment option should be determined according to the centre’s experience. Anyway, in case of complete arterial occlusion the 2-h time limit for revascularisation must be considered: indeed, after 2 h of complete renal ischaemia also a good technical result will bring a poor functional outcome. Nephrectomy is always recommended if conservative treatments fail [45].

**Fig. 11:**
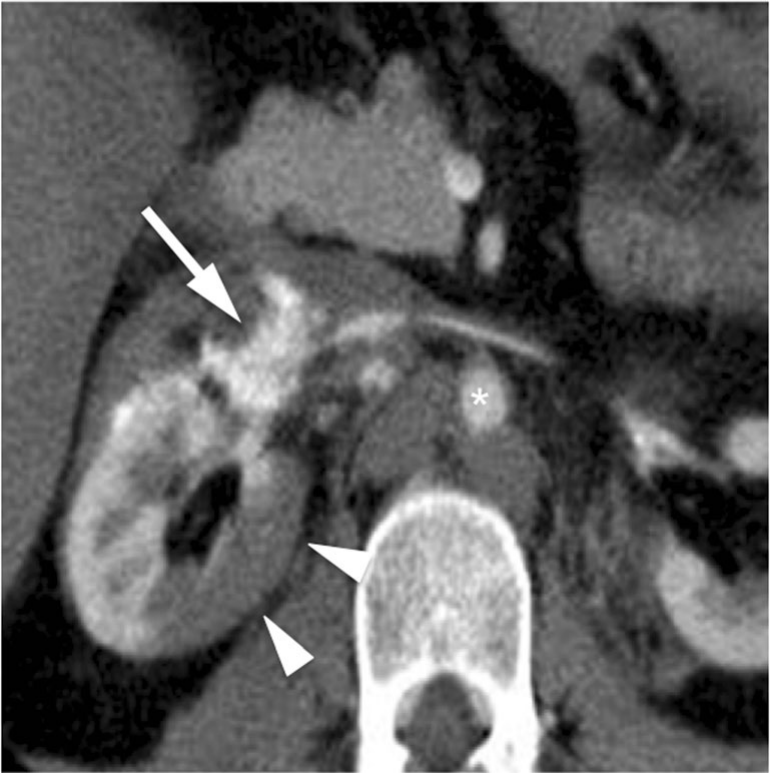
Main renal artery laceration (AAST grade IV). On this 3-mm-thick multiplanar reconstruction (*MPR*) axial image acquired during the arterial (**a**) phase of the study, a large contrast material extravasation can be observed adjacent to the *right* renal artery (*arrow*) as a consequence of renal artery laceration; to notice, the markedly reduced diameter of the aorta (*star*) as a consequence of the haemodynamic shock status. Moreover, the right renal parenchyma shows a large well-defined hypodense area (*arrowheads*), representing renal infarction

## Conclusions

In case of renal trauma, MDCT can generate a wide spectrum of findings that a radiologist must be able to recognise in order to correctly direct trauma management and allow accurate risk stratification. Anyway, it is important to notice that, in the emergency setting, the differentiation between AAST grade 1, 2 and 3 renal injuries does not result in a change in patient management, the “wait-and-see” approach being the most indicated in all cases of haemodynamic stability. On the other hand, AAST grade 4 and 5 lesions must be promptly recognised and communicated to the clinician because they will shift the treatment to an endourological, endovascular or surgical one; indeed, the simple “wait-and-see” management may be considered only in case of segmental infarctions involving less than 50 % of the renal parenchyma and of shattered kidney without urine or active blood extravasation. Moreover, CT may highlight some peripheral vascular lesions (i.e., active bleedings, pseudoaneurysms and artero-venous fistulae) that are not directly mentioned in the AAST classification, but that must be accurately recognised because of requiring, in the majority of the cases, an endovascular interventional approach.

## Abbreviations

AAST: American Association for the Surgery of Trauma
NOM: Non-operative management
CT: Computed tomography
MDCT: Multidetector computed tomography
CEUS: Contrast-enhanced ultrasound
MPR: Multiplanar reconstruction
MIP: Maximum intensity projection
VR: Volume rendering
HU: Hounsfield units
